# Implicit Motives as Determinants of Networking Behaviors

**DOI:** 10.3389/fpsyg.2018.00411

**Published:** 2018-04-30

**Authors:** Hans-Georg Wolff, Julia G. Weikamp, Bernad Batinic

**Affiliations:** ^1^Department of Psychology, Faculty of Humanities, University of Cologne, Cologne, Germany; ^2^Department of Psychology, University of Würzburg, Würzburg, Germany; ^3^Department of Education and Psychology, Johannes Kepler University of Linz, Linz, Austria

**Keywords:** networking, social capital, implicit motives, work behavior, social interaction, career self-management

## Abstract

In today’s world of work, networking behaviors are an important and viable strategy to enhance success in work and career domains. Concerning personality as an antecedent of networking behaviors, prior studies have exclusively relied on trait perspectives that focus on how people feel, think, and act. Adopting a motivational perspective on personality, we enlarge this focus and argue that beyond traits predominantly tapping social content, motives shed further light on instrumental aspects of networking – or why people network. We use McClelland’s implicit motives framework of need for power (nPow), need for achievement (nAch), and need for affiliation (nAff) to examine instrumental determinants of networking. Using a facet theoretical approach to networking behaviors, we predict differential relations of these three motives with facets of (1) internal vs. external networking and (2) building, maintaining, and using contacts. We conducted an online study, in which we temporally separate measures (*N* = 539 employed individuals) to examine our hypotheses. Using multivariate latent regression, we show that nAch is related to networking in general. In line with theoretical differences between networking facets, we find that nAff is positively related to building contacts, whereas nPow is positively related to using internal contacts. In sum, this study shows that networking is not only driven by social factors (i.e., nAff), but instead the achievement motive is the most important driver of networking behaviors.

## Introduction

Roughly 20 years ago, theories on boundaryless and protean careers (e.g., Arthur and Rousseau, 1996; Hall, 1996) suggested that the responsibility for individuals’ careers has shifted from the organization to the individual. This shift has increased the importance of career self-management behaviors, such as networking, visibility, and positioning behaviors, or individual investments in human capital (Sturges, 2008). Networking refers to building, cultivating, and utilizing informal relationships to assist in people’s work or career (e.g., Forret and Dougherty, 2004; Gibson et al., 2014), and studies have shown that it is positively related to salary (Forret and Dougherty, 2004; Blickle et al., 2009; Wolff and Moser, 2009), promotions (Michael and Yukl, 1993; Wolff and Moser, 2010), work performance (Blickle et al., 2012), success as an entrepreneur (Witt, 2004), and getting a job (Van Hoye et al., 2009). In the light of these positive effects of networking on work and career outcomes, scholars have also examined determinants of networking to gain further insights into the origins of this construct. This research has shown that contextual variables such as job level (Michael and Yukl, 1993; Ng and Feldman, 2011) or managerial function (Michael and Yukl, 1993; Forret and Dougherty, 2001) as well as personality variables such as extraversion, proactivity, or self-esteem (Wanberg et al., 2000; Forret and Dougherty, 2001; Lambert et al., 2006) affect networking behaviors. In addition, scholars found that determinants are differentially related to specific networking dimensions (Forret and Dougherty, 2001; Ng and Feldman, 2010; Wolff and Kim, 2012), for example, managerial function (i.e., marketing vs. accounting and production) is associated with external, but not internal networking behaviors, respectively (Michael and Yukl, 1993).

While this research has enhanced our understanding of networking behaviors, our knowledge on the relationship between personality and networking appears limited, as the motivational perspective of personality – *why* people network – is largely missing. Research on personality falls into two distinct research perspectives (e.g., McAdams and Olson, 2010), the lexical trait perspective (e.g., McCrae and Costa, 1997) and the motivational perspective (e.g., Schultheiss et al., 2012). Prior research has exclusively adopted a trait perspective that refers to descriptive dimensions of *how* individuals act, think, and feel in a general manner (Goldberg, 1990; McCrae and Costa, 1997; Ashton and Lee, 2001). These studies have found that networking is related to traits tapping how people interact with others (e.g., self-monitoring, Ferris et al., 2008; extraversion and agreeableness, Wanberg et al., 2000) and how they approach tasks (e.g., proactivity, Thompson, 2005). However, studies remain silent with regard to a motivational perspective and the relationship of fundamental needs or motives with networking. There is a lack of knowledge on how personality contributes to the goals “networkers” seek to attain, and what needs people seek to satisfy by networking. We suggest that implicit motives predispose people to consistently show specific spontaneous affective and behavioral reactions that facilitate networking and thus increase the likelihood of networking behaviors. In fact, networking has been anecdotically mentioned in association with a variety of motives in the literature, the need for power (nPow), *and* the need for affiliation (nAff) (Kehr, 2004a; Porter and Woo, 2015) or all three needs (Steinmann et al., 2016), whereas Winter et al. (1998) suggest that individuals with a low nAff might network. Therefore, scholars have recently called for research on motivational and need theories to extend our knowledge of what drives individuals to engage in networking behaviors (Casciaro et al., 2014; Porter and Woo, 2015, p. 19).

To further our knowledge on how personality affects networking, we adopt a motivational framework linking networking to McClelland’s (1987) theory on implicit motives. Specifically, we focus on the prominent “Big Three” (Schultheiss and Brunstein, 2001, p. 72) motives of need for achievement (nAch), nPow, and nAff. Using this framework, we adopt a more instrumental perspective on personality and argue that networking is not only shown for social reasons unraveled by the trait perspective, but is also shown for instrumental reasons, such as career success and work performance. In contrast to the anecdotal presumptions (see previous paragraph) that mostly pertain to the nAff and nPow, we suggest that networking is a means to satisfy the nAch, because the essential goal of networking is to excel in work or career domains. As the nAch is also the driver of entrepreneurial behavior (McClelland, 1987; Rauch and Frese, 2007), we essentially propose that “Networkers” are truly self-managers – or entrepreneurs – of their careers. In addition, we examine how the three motives relate to facets (or subdimensions) of networking. Scholars (e.g., Gibson et al., 2014; Porter and Woo, 2015) depict networking as a multifaceted construct and we examine how the three motives relate to (1) internal vs. external networking and (2) building, maintaining, and using contacts (Wolff et al., 2011). We suggest that the nAch is broadly related to all networking facets, whereas the needs for power and affiliation are differentially related to specific, but not all facets of networking.

Our study contributes to research on antecedents of networking in several ways. First, as scholars argue that, in the light of organizational changes in the past decades (Allred et al., 1996), networking has gained in importance for individual (Hall, 1996) as well as organizational success (Pentland, 2012), our study advances our knowledge on an important organizational behavior. We heed recent calls (Porter and Woo, 2015) and supplement the trait perspective on determinants of networking by adding a motivational perspective, contributing to a broader, more complete picture of the links between networking and personality. Also note that we broaden this picture with our focus on implicit constructs whereas prior studies examined explicit (i.e., consciously accessible) constructs. Implicit motives are represented as unconscious associative networks and unconsciously affect cognition, affect, and behavior, whereas explicit concepts are consciously accessible and based on deliberative thinking (McClelland et al., 1989; Kehr, 2004b). While human behavior is often governed by conscious goals, people do not always deliberate upon their actions (Bargh and Chartrand, 1999) and how they relate to their various goals: People often act spontaneously and sometimes even against their deliberately set goals (e.g., the failure to stick to new year’s resolutions). Second, the motivational perspective allows a more instrumental view on the relationship between personality and networking. Personality affects networking not only due to trait differences in social or task orientation. We contend that differences in motives also affect whether people use networking as a means to goal attainment and need satisfaction. We emphasize career related, entrepreneurial-like activities and thus advance knowledge on why people show networking, an important career strategy. Third, we also contribute to our knowledge on differential relations of networking facets with personality variables. While most research has examined differences between internal and external networking (e.g., MacCallum et al., 2014), we focus on the stages of building, maintaining, and using contacts, and thus put Wolff and Kim’s (2012) suggestions of social and instrumental differences of networking facets to an additional test. Finally, knowledge on the relationship between implicit motives and networking is valuable to practitioners who aim to attract networkers to certain jobs or who stage networking events. Understanding why individuals differ in their networking behaviors is also important for practitioners engaged in networking trainings (de Janasz and Forret, 2008) as well as organizations in their quest for effective teamwork (Pentland, 2012) or the implementation of network-building HR practices (Collins and Clark, 2003).

### Networking

Research on networking is rooted in three streams of research. First, theories on protean and boundaryless careers (Arthur and Rousseau, 1996; Hall, 1996) argue that organizations have changed from hierarchical, bureaucratic structures to delayered, more networked structures (Allred et al., 1996; Arthur and Rousseau, 1996). As a result, organizations have cut their responsibility for employees’ careers and this has heightened the importance of career self-management, for example, by means of networking (King, 2004; Sturges, 2008). In this vein, studies show that networking is beneficial for a wide range of career outcomes (e.g., Forret and Dougherty, 2004) and also for work performance (Thompson, 2005; Blickle et al., 2012). In a second research stream, networking abilities represent an important component of political skills (e.g., Ferris et al., 2007; Blickle et al., 2012). As individuals in organizations struggle for power and control over scarce resources, political tactics, such as persuasion, sanctions, or networking are informal means to secure one’s share (Pfeffer, 1981). This political perspective suggests that individuals use networking to influence others (Zanzi et al., 1991), to get things done (Kotter, 1982), and to get ahead (Luthans et al., 1985). A third stream has evolved in research on entrepreneurship. The identification and exploitation of entrepreneurial opportunities is embedded in a social context and attaining resources such as information, ideas, or venture capital promote entrepreneurial success (e.g., Brüderl and Preisendörfer, 1998; Witt, 2004). The time entrepreneurs invest into networking or the size of their networks positively affects their venture’s performance (Semrau and Sigmund, 2012).

In all three research streams, scholars broadly define networking as individuals’ behaviors aimed at building, maintaining, and using work-related contacts in order to obtain resources, such as influence or support in their work and careers (Forret and Dougherty, 2001; Witt, 2004; Ferris et al., 2005). Examples of networking behaviors include asking others for informal advice on work-related matters, going out for drinks with colleagues, participating in professional activities, or staying in contact with former colleagues to stay up to date in business matters. The benefit of networking contacts rests on their particularistic (vs. universalistic) character, that is, individuals obtain access to resources because they have developed a personal, trusting relationship with a contact. In this vein, networking predominantly refers to informal contacts that are not part of the formal organizational structure (Michael and Yukl, 1993). Scholars also describe networking contacts as cooperative, emphasizing the importance of reciprocity in the exchange of resources and favors (Cohen and Bradford, 1989; Bozionelos, 2003).

#### Networking Dimensions

Several scholars suggest that networking consists of multiple dimensions or facets (Forret and Dougherty, 2001) that can be subsumed under a broad, higher-order construct of networking (Ferris et al., 2005). Support for this hierarchical structure comes from several studies that report differential relations between networking dimensions and its determinants (Michael and Yukl, 1993; Ng and Feldman, 2010; Wolff and Kim, 2012) as well as its outcomes (Forret and Dougherty, 2004; Porter et al., 2016). In the present study, we use a multidimensional networking model introduced by Wolff et al. (2011), as it subsumes several other models and has been used in prior theorizing on determinants of networking behaviors (Wolff and Muck, 2009; Wolff and Kim, 2012). In particular, we use the functional facet described in the subsequent paragraph to hypothesize differential relations between networking facets and motives.

Wolff et al.’s (2011) and Wolff and Kim’s (2012) networking model consists of two facets. First, in line with other networking models (Michael and Yukl, 1993; Ng and Feldman, 2010; Andrews et al., 2011), a structural facet distinguishes internal from external networking, referring to contacts within and outside one’s organization, respectively. Wolff and Kim (2012) argue that contacts differ along this distinction in range and accessibility. In comparison to internal contacts, where accessibility is high (e.g., simply dropping by one’s office, cf. Kotter, 1982) and range is limited due to higher homogeneity within an organization, external contacts are typically less accessible (e.g., scheduling when to meet and traveling), but the range of contacts is much less restricted. For example, outside their organization, individuals can network with individuals from other industry sectors, from politics, or from other countries.

The second facet of Wolff et al.’s (2011) model refers to a functional distinction of building, maintaining, and using contacts that depicts the prototypical process of relationship development. Networking behaviors are thus not restricted to “meeting new people” (Gibson et al., 2014, p. 149; see also Porter and Woo, 2015). Wolff and Kim (2012) argue that along these stages of relationship development, social concerns become less important, whereas instrumental concerns become more important. Building contacts is a genuinely social behavior and might serve multiple goals, for example, simple pleasure and entertainment as well as the development of business contacts. Also, when individuals meet new contacts, instrumentality remains vague as they cannot fully judge whether a person possesses valued resources and will become an important contact. With regard to maintaining contacts, instrumental concerns become more important and supplement or even outweigh social concerns, for example, when individuals decide to follow up a person they met. In using contacts, instrumental concerns clearly predominate as individuals are actively approaching a contact in order to obtain a needed resource. Even though using contacts is embedded in a social interaction, the attainment of resources is the very reason for initiating the interaction.

#### Determinants of Networking Behaviors

To further examine the origins of networking behaviors, research on determinants of networking has examined contextual as well as individual variables. Studies on contextual determinants have postulated that specific job characteristics, such as dependency on others to fulfill job tasks (Michael and Yukl, 1993) or an enterprising job context that requires more interpersonal skills (Blickle et al., 2012), affect networking. In this vein, individuals in supervisor (vs. non supervisor) positions (Michael and Yukl, 1993; Forret and Dougherty, 2001; Ng and Feldman, 2011) as well as individuals in marketing (vs. production or finance) positions show more networking (Michael and Yukl, 1993; Kuipers and Scheerens, 2006, only for external networking).

With regard to personality, research has examined traits that tap into domains of social behaviors or how people approach their work tasks. Here, we define the term trait as “comparative features of psychological individuality that account for consistencies in behavior, thought, and feeling across situations and over time” (McAdams and Olson, 2010, p. 519). The most prominent “Big Five” model (McCrae and Costa, 1997) is based on a lexical perspective, that is, factors were derived from analyses of long lists of adjectives used in everyday language to describe individuals (e.g., Goldberg, 1990). Traits thus refer to habitual patterns of how people behave in general. With regard to networking, several studies found that it is positively related to traits tapping social behaviors such as extraversion and agreeableness (Wanberg et al., 2000; Forret and Dougherty, 2001; Van Hoye et al., 2009; Wolff and Kim, 2012) and also to self-monitoring (Ferris et al., 2005; Wolff and Moser, 2006). Using the interpersonal circumplex, Wolff and Muck (2009) showed that networking is related to both, the dominance and the warmth dimensions. With regard to traits describing how people approach tasks, scholars report positive associations between networking and proactivity (Thompson, 2005; Lambert et al., 2006; Ferris et al., 2008), self-efficacy (Ferris et al., 2008), or locus of control (Ferris et al., 2005; Ng and Feldman, 2011). In sum, “networkers” can be characterized as social and self-starting individuals.

Next to these relationships between traits and networking in general, some studies have also related traits to the structural and functional distinctions of networking behaviors to examine differential relations between traits and networking. Concerning the structural distinction, findings indicate that internal networking is more strongly associated with extraversion and agreeableness due to the higher accessibility and proximity of internal contacts (Wolff and Muck, 2009; Wolff and Kim, 2012). In addition, external networking is more strongly associated with self-monitoring (Wolff and Moser, 2006) and dominance (Wolff and Muck, 2009), presumably because of the higher relevance of adaptability to a range of different situations and the necessity to expend more effort (i.e., to be proactive). Not many studies have examined differential relations for the functional distinction of building, maintaining, and using contacts. These show that dominance is more important in building contacts (Wolff and Muck, 2009), whereas agreeableness appears to be irrelevant to building contacts (Wolff and Kim, 2012).

### Motives

Next to trait perspectives, the motivational perspective represents a distinct framework to describe individual differences in personality (Winter et al., 1998; McAdams and Olson, 2010). According to Winter, this distinction goes back on the works of Allport and Murray who examined traits and motives, respectively. While traits refer to habitual patterns of *how* people think, feel, and act, the motivational perspective focuses on *why* people act and describes personality in terms of individual differences in motives. Motives are defined as the “capacity to experience a specific type of incentive as pleasurable” (Schultheiss et al., 2012, p. 652) and thus – with regard to personality – refer to stable differences in classes of goals and desires that drive individual behavior (McClelland, 1987). Motives affect the perception and interpretation of situations by selecting, energizing, and regulating behavior and also serve to evaluate goal states and the probability of success in a situation. As motives refer to individual goals, they are distinct from traits that refer to habitual patterns of behavior, essentially ignoring why individuals show this behavior (McClelland, 1987; Winter et al., 1998; Lang et al., 2012). For example, Winter et al. (1998) suggest that extraverts might attend parties to fulfill affiliative needs, but introverts might attend parties to pursue other goals, “such as networking for a job” (p. 235).

An important distinction is between implicit and explicit motives. Explicit motives refer to self-attributed needs that are consciously accessible and can be assessed in questionnaires based on standard self-ascriptions. Implicit motives represent unconscious associative networks (Kehr, 2004a) of idealized self-conceptions (Winter et al., 1998) that can be assessed by projective measures like the Thematic Apperception Test (TAT). Implicit motives develop in early childhood (McClelland et al., 1989) and predict long-term spontaneous behavioral trends over time (Spangler, 1992). Thus, implicit and explicit motive systems have different scopes, can be activated differently, correlate only weakly to moderately, and predict different aspects of behavior (McClelland, 1987; McClelland et al., 1989; Sokolowski et al., 2000; Kehr, 2004b; Schultheiss et al., 2012). In this paper, we focus on the “Big Three” (Schultheiss and Brunstein, 2001, p. 72) motives, the nAch, the nPow, and the nAff.

The nAch describes the striving to do things better. nAch is activated in situations when people are confronted with criteria of excellence, competence, or efficiency (McClelland, 1987; Langens and Schmalt, 2008). The goals of people high in nAch are high-task performance and efficiency in task accomplishment, as opposed to working hard in itself (McClelland and Franz, 1992). Individuals high in nAch take moderate risks and value feedback on their performance. They are also innovative and more active in their search for information (McClelland, 1987). With regard to the work domain, the achievement motive is closely linked to occupational success, for example, salary and promotions (McClelland and Franz, 1992; Winter, 2010), entrepreneurial success (Collins et al., 2004), and sales growth (Chuzmir and Azevedo, 1992).

The affiliation motive describes the desire to be with other people (McClelland, 1987). Individuals high in nAff are sensitive to situations in which they interact with friends as well as strangers. In social interaction, their goal is to establish and maintain positive social relationships. Accordingly, they prefer cooperation and compromise over conflict (Langner and Winter, 2001; Winter, 2007). Individuals high in nAff also tend to choose friends over experts as working partners and are particularistic rather than universalistic in applying rules, sometimes “to the detriment of organizational requirements” (House et al., 1991, p. 367). In management positions, individuals high in nAff have good relations with their subordinates (Steinmann et al., 2016), but avoid tough decisions and conflict. Yet, they perform well as managerial integrators when performance depends upon getting individuals together and resolving their differences (McClelland, 1987). In fact, some scholars suggest that nAff might have become more important in recent times due to the organizational and career changes described in the section “Introduction” (Steinmann et al., 2016).

The nPow describes the need to have social, physical, or emotional impact (McClelland, 1987; Schultheiss et al., 2012). It is activated in situations where individuals can control or influence others (Langens and Schmalt, 2008). Individuals high in nPow aim for prestige and reputation (McClelland, 1987). They are good at organizing and coordinating others (McClelland, 1987; Steinmann et al., 2015) and are effective in persuading others (Schultheiss and Brunstein, 2002). In this sense, nPow pertains to the core of organizational functioning. However, people high in nPow do not hesitate to break up coalitions, they are confrontational in negotiations and rarely have the ability to share and listen to others (McClelland, 1987). The nPow is prominently associated with leadership and success in politics and business. With regard to US Presidents, for example, scholars found that those high in nPow are higher in presidential charisma (House et al., 1991) and perceived greatness, but are also more likely to enter wars (Winter, 1987; Spangler and House, 1991). With regard to the business domain, nPow is associated with advancement to positions, whenever managing and influencing others rather than working at specific tasks is vital [McClelland, 1987; but see Winter (2010) for a different account]. In this vein, the nPow is positively associated with movement into top management (McClelland and Boyatzis, 1982; Jacobs and McClelland, 1994), Charisma of CEOs (De Hoogh et al., 2005), profit growth (Chuzmir and Azevedo, 1992), and management of more important subsidiaries (Cornelius and Lane, 1984).^1^

### Networking and Implicit Motives

Here, we suggest that some motives are related to networking in general, whereas other motives are only related to specific facets of networking behaviors. Specifically, we argue that nAch positively affects networking behaviors in general, whereas the effects of nAff and nPow depend on the functional facet of building, maintaining, and using contacts. Our general premise is that implicit motives facilitate networking for three reasons. First, people high in a specific need show networking behaviors as a means to satisfy this need in a wider range of situations due to easier arousal of the need by situational cues, resulting in a higher frequency of networking behaviors. Second, specific needs energize behavior, resulting in higher frequency and persistence of networking behavior. Third, people high in relevant needs more likely habitualize networking behaviors and this should increase the availability of behavioral networking scripts and decrease the amount of conscious willpower (i.e., volition) required to show networking behavior (Kehr, 2004b).

Concerning the achievement motive, we predict a general relation with networking behaviors. People high in nAch strive for success in their work tasks, their careers, or entrepreneurial success. To obtain these goals, individuals high in nAch resort to networking, because it yields valuable resources and promotes goal attainment. Note that typically nAch is associated with a focus on individual work tasks in laboratory studies, because persistence or effort yields success in such tasks. Yet, the nAch is not satisfied by expending effort or persisting. These are merely means to attain success, and people high in nAch use many means to attain highly valued outcomes (McClelland and Franz, 1992; Winter, 2010). We suggest, that today’s complex, interdependent work tasks and also career paths within and across organizations require social means to attain success – and people high in nAch will resort to these means as they promise success (French and Lesser, 1964). For example, informal task advice obtained by networking improves performance and strategic information allows a more realistic judgment of tasks or promotion options and thus the probability of success at work or in their career. Because resource acquisition and success is tied to the full process of building, maintaining, and using contacts, we assume a general relation between nAch and networking facets.

Hypothesis 1: The achievement motive is positively related to networking behaviors.

With regard to the affiliation motive, we assume that this desire for social interactions facilitates building and maintaining contacts because of the social content of these facets. According to Wolff and Kim (2012), the social (vs. instrumental) content of networking is higher in building contacts, gradually decreases in maintaining contacts, and even more so in using contacts, whereas instrumental content increases reciprocally. Individuals high in nAff who enjoy social interactions for the sake of interacting therefore satisfy their goals by means of the social (i.e., building and maintaining contacts) as opposed to the instrumental (i.e., using contacts) aspects of networking behaviors. They focus on people, not performance (Schultheiss et al., 2012), and need satisfaction is not tied to resource attainment from using contacts. In addition, individuals high in affiliation have been characterized as cooperative (Langner and Winter, 2001), a feature that many definitions of networking emphasize, as well. People high in affiliation therefore do favors to others and act in a particularistic manner, but fail to strategically manage their contacts: they do not take the instrumental value of their contacts into account.

Hypothesis 2: The affiliation motive is positively related to building and maintaining contacts.

With regard to the power motive, we predict a positive association between this motive and using contacts, but not building and maintaining contacts. In line with the political perspective on networking (Pfeffer, 1981; Ferris et al., 2007), individuals high in nPow will use their contacts to attain resources and influence, or to protect their power. In fact, some ways of using contacts, for example, gathering support for personal agendas might directly satisfy the nPow. However, we also suggest that individuals high in nPow mainly focus the instrumental aspects of networking and tend to overlook that in networking relationships instrumentality (i.e., the exchange of resources) rests upon trusting relationships with others rather than mere resource dependency (Pfeffer, 1981). In this vein, the interpersonal style of individuals high in nPow does not appear to fit building and maintaining contacts. For example, Winter (2007) suggests that individuals high in power motivation are confrontational and exploitative in negotiations, whereas cooperation and the (presumably fair) exchange of resources are a core feature of networking. In this vein, McClelland (1987) also reports that individuals high in power motivation break up coalitions in order to take advantage of their partners.

Hypothesis 3: The power motive is positively related to using contacts.

## Materials and Methods

### Procedure and Participants

We collected data using a commercial online access panel (see e.g., Callegaro et al., 2014) whose members have agreed to regularly participate in survey and market research. We assessed motives and networking behaviors 2 weeks apart to minimize common method bias (Podsakoff et al., 2012). At Time 1, we invited 1,717 participants and collected data on implicit motives from 1,003 participants (response rate: 58%). At Time 2, we invited 996 individuals (reduction of seven participants due to invalid email addresses) again and obtained data on networking from 684 participants (response rate: 69%). Of these, we excluded participants for three reasons. First, we excluded 24 participants for insufficient effort responding (e.g., Malhotra, 2008; Huang et al., 2012). Specifically, we followed Huang et al. (2012) who suggested that excluding individuals who expend <2 s per item to complete a survey is a conservative approach with “high specifity while detecting a reasonable proportion of IER [insufficient effort responding] protocols” (p. 111). Rounding to minutes, this resulted in a lower cutoff of 3 (exact number would have been 206 s) and 2 min (106 s) for the motive (excluding three persons) and networking surveys (excluding two), respectively. We also excluded participants those who took >30 min to complete either survey (7 persons at T1 and 11 at T2), because this indicates interruptions in survey completion and might indicate insufficient effort, as well. Second, in line with prior studies (e.g., Forret and Dougherty, 2004; Wolff and Kim, 2012), we excluded 96 participants who were not employed full time (i.e., self-employed or free-lancers as well as those working <20 h per week), because due to differences in networking opportunities their networking behaviors might differ. Finally, we excluded 25 individuals with missing data in our study variables for a final sample size of *N* = 539.

Of these participants, 297 (55%) were female, 242 (45%) were male, with a mean age of 37.0 years (*SD* = 10.20). According to the international standard classification for education (UNESCO Institute for Statistics, 2012), the majority of respondents (38%) had completed tertiary education (i.e., university degree), 29% had completed upper secondary education (i.e., 13 years of schooling) and 33% had completed lower secondary education (i.e., 9 or 10 years of schooling). The average organizational tenure was 8.3 years (*SD* = 8.5) and 116 individuals (22%) indicated that they were in a supervisor position.

### Measures

#### Motives

We used the Multi-Motive Grid [MMG, Schmalt et al. (2000), referred to as the short version in Sokolowski et al., 2000] to access the implicit system and assess the achievement, affiliation, and power motive. The MMG is a published test and has been used in a range of studies in the work or careers domain (Abele et al., 1999; Sokolowski et al., 2000; Kehr, 2004a; Lawrence and Jordan, 2009). Langens and Schmalt (2008) provide an overview on the validity and findings reported for this measure showing that it predicts similar criteria as the TAT. Because no scoring of stories is required for the MMG, it is more economic and allowed us to examine a bigger sample and thus attain higher power. Similar to the TAT, the MMG assesses implicit motives by means of pictorial stimuli (Bilsky and Schwartz, 2008). However, instead of response assessment by story writing, the MMG uses statements representing motivational tendencies (e.g., “anticipate to loose standing” or “feeling good about meeting other people” for power and affiliation, respectively) and participants indicate whether these statements fit the picture (0 = no, 1 = yes). The MMG uses 14 pictures, with a total of 12 statements representing approach and avoidance tendencies of the three motives. The scales represent the nAch (i.e., hope of success; α = 0.71 and fear of failure α = 0.66), the nAff (i.e., hope of affiliation α = 0.63, fear of rejection, α = 0.76), and the nPow (i.e., hope of power α = 0.80, fear of power, α = 0.71).^2^ To obtain scores for the needs for power, achievement, and affiliation, we calculated net motives (also labeled resultant motives, e.g., by Langens and Schmalt, 2008), subtracting the standardized fear score from the standardized hope score for each motive. This is in line with prior research (Sokolowski et al., 2000; Kehr, 2004a) and also longstanding practice in motivation research (Heckhausen, 1968). For example, McClelland (1987, p. 564) reports that a reanalysis of his own data by Heckhausen (1968) using net motives yields results similar to the usual TAT scoring system and represents theoretical predictions more clearly than either hope or fear components alone. Because net motives are calculated from two separate scales that measure related, but distinct concepts, we used formulas provided by Nunnally (1978, p. 246) to calculate composite reliabilities for the net motive scores. They were all satisfactory with reliabilities of 0.83, 0.73, and 0.78 for the nPow, nAch, and nAff, respectively.

#### Networking

We assessed networking using Wolff and Moser’s (2006, Wolff et al., 2011) multidimensional 44-item questionnaire. Porter and Woo (2015) suggest that their networking model is among the most elaborate models and several studies provide evidence for its validity (e.g., Wolff and Moser, 2006) and its potential to uncover differential relationships with personality (e.g., Wolff and Kim, 2012). In accordance with the two facets of (1) internal vs. external networking and (2) building – maintaining – using contacts, this measure consists of six subscales. These subscales are building internal contacts (six items, e.g., “I use company events to make new contacts”; α = 0.82), maintaining internal contacts (eight items, e.g., “I catch up with colleagues from other departments about what they are working on”; α = 0.79), using internal contacts (eight items, e.g., “I use my contacts with colleagues in other departments in order to get confidential advice in business matters”; α = 0.81), building external contacts (seven items, e.g., “I accept invitations to official functions or festivities out of professional interest”; α = 0.83), maintaining external contacts (seven items, e.g., “I ask others to give my regards to business acquaintances outside of our company”; α = 0.83), and using external contacts (eight items, e.g., “I exchange professional tips and hints with acquaintances from other organizations”; α = 0.85).

We considered several potentially relevant control variables, in particular age, education, gender, and several organizational variables (e.g., supervisor position and tenure). While we report the correlations between these and our substantive variables in **Table 1**, we decided not to include them in our analyses for several reasons. First, the benefits of including controls has recently been questioned (e.g., Becker, 2005) and some have argued that controls should be left out unless strong theoretical assumptions suggest to include them (Bernerth and Aguinis, 2016). This does not apply in the present case. Rather, on theoretical grounds motives represent stable dispositions acquired well before individuals receive an education or enter the workforce, and motives are determinants of educational and vocational decisions (McClelland, 1987; McClelland and Franz, 1992). Due to this temporal sequence, our potential control variables can hardly causally affect the relation between motives and networking, but rather might partial “true variance,” and result in underestimation of true effects (Spector et al., 2000). Moreover, analyses including controls did not alter conclusions substantively.

**Table 1:** Descriptive statistics and correlations of study variables.

	*M*	*SD*	1	2	3	4	5	6	7	8	9	10	11	12	13
(1) Age	36.98	10.20	-												
(2) Gender	1.55	0.50	-0.20**	-											
(3) Education	3.58	1.38	-0.11**	-0.05	-										
(4) Org. tenure	8.30	8.50	0.57**	-0.20**	-0.17**	-									
(5) Supervisor position	1.78	0.42	-0.12**	0.09*	0.09*	0.08	-								
(6) Need for power	0.00	1.10	0.16**	-0.10*	-0.14**	0.07	-0.04	-							
(7) Need for affiliation	0.00	1.19	0.16**	-0.07	-0.09*	0.06	-0.08	0.42**	-						
(8) Need for achievement	0.00	1.27	0.04	-0.05	-0.06	0.03	-0.04	0.44**	0.28**	-					
Internal networking															
(9) Building contacts	2.22	0.59	0.04	0.01	0.05	-0.05	-0.21**	0.17**	0.20**	0.20**	-				
(10) Maintaining contacts	2.52	0.50	0.02	0.02	0.09	-0.03	-0.18**	0.15**	0.13**	0.19**	0.52**	-			
(11) Using contacts	2.37	0.54	-0.14**	0.06	0.05	-0.08	-0.05	0.15**	0.12**	0.16**	0.42**	0.61**	-		
External networking															
(12) Building contacts	1.72	0.56	0.10*	-0.11*	0.11*	0.02	-0.26	0.14**	0.16**	0.17**	0.52**	0.42**	0.41**	-	
(13) Maintaining contacts	1.88	0.57	-0.16**	-0.01	0.13**	-0.12**	-0.12**	0.09*	0.09*	0.16**	0.42**	0.50**	0.55**	0.65**	-
(14) Using contacts	1.90	0.56	-0.23**	0.08	0.15**	-0.24**	-0.07	-0.02	-0.02	0.05	0.30**	0.39**	0.55**	0.41**	0.62**

*N = 539. Gender (1 = male, 2 = female), education (1 = 9 years of schooling, 2 = 10 years, 3 = 12 years, 4 = 13 years, 5 = university degree), and supervisor position (1 = yes, 2 = no) are categorical variables. ^∗^*p* < 0.05; ^∗∗^*p* < 0.01*.

#### Analyses

We analyzed our hypotheses by means of latent multivariate regression simultaneously regressing the six networking facets on the three motives using Lisrel 8.72 (Jöreskog and Sörbom, 2005). Specifically we used a single indicator structural equation model accounting for the unreliability of the scales by fixing the variance of each manifest variable to one minus the reported reliability multiplied by the variance of the respective scale. In a first step, we examined our hypothesized model defined by hypotheses 1–3, restricting other paths to zero. We use Chi-squared tests to examine model fit and *t*-values (one-tailed tests) to examine significance of path coefficients. In a second exploratory step, we sought to improve our model by examining modification indices, insignificant coefficients, and standardized residuals.

## Results

**Table 1** shows descriptive statistics and zero-order correlations of the study variables. **Table 2** shows path coefficients from our multivariate regression model. Overall, the model fit very well (χ^2^(6) = 3.31, *p* = 0.78).

**Table 2:** Results from multivariate latent regression of networking behaviors on motives: hypothesized model.

	Internal networking			External Networking		
	Building contacts	Maintaining contacts	Using contacts	Building contacts	Maintaining contacts	Using contacts
nAch	0.20^∗∗^	0.23^∗∗^	0.15^∗^	0.17^∗∗^	0.18^∗∗^	0.11^∗^
nAff	0.16 ^∗∗^	0.07	–^a^	0.12^∗^	0.04	–^a^
nPow	–^a^	–^a^	0.10^∗^	–^a^	–^a^	-0.08

*N = 539. A single indicator latent variable model is used, coefficients from standardized solution are shown. χ^*2*^(6) = 3.31, *p* = 0.78. nAch, nAff, and nPow refer to the needs of achievement, affiliation, and power, respectively. ^∗^*p* < 0.05; ^∗∗^*p* < 0.01 (one-tailed). ^*a*^Coefficient restricted to zero*.

Hypothesis 1 predicts a general association of nAch with networking behaviors. In support of this hypothesis, all six path coefficients are significant and in the predicted direction. Hypothesis 2 proposes that nAff is positively associated with building and maintaining contacts. This assumption receives partial support, as nAff significantly predicts building internal as well as external contacts, but not maintaining internal and external contacts. Finally, Hypothesis 3 predicts an association of nPow with using contacts. Again, this hypothesis receives partial support as nPow is significantly associated with using internal, but not with using external contacts. In fact, the latter relation is negative, though not significant.

We estimated several additional models to further examine the relationships in an exploratory manner. We first examined standardized residuals and modification indices, but they did not indicate the necessity to include additional paths into our model (i.e., all residuals <1 and modification indices <2). We then restricted the insignificant coefficients of nAff to maintaining (internal and external) contacts to zero. The resulting model (model 2) fit well [χ^2^(8) = 5.46, *p* = 0.71] and not significantly worse than our hypothesized model [Δχ^2^(2) = 2.15, *p* = 0.34]. As in this model, the coefficients of nPow to using contacts were only marginally significant (i.e., at *p* < 0.10), we also examined a model (model 3) restricting these two coefficients to zero. While this model still fit the data well [χ^2^(8) = 13.41, *p* = 0.20] it fit significantly worse than the previous model [Δχ^2^(2) = 7.95, *p* = 0.018]. We therefore decided to retain Model 2. The coefficients of this model are depicted in **Table 3**. nAch is consistently related to all networking facets, and nAff is related to building internal as well as external contacts. The effect of nPow on using internal contacts only approaches significance in this model (*p* = 0.058).

**Table 3:** Results from multivariate regression of networking behaviors on motives: final model following exploratory model respecification.

	Internal networking			External networking		
	Building contacts	Maintaining contacts	Using contacts	Building contacts	Maintaining contacts	Using contacts
nAch	0.21^∗∗^	0.26^∗∗^	0.17^∗^	0.19^∗∗^	0.20^∗∗^	0.12^∗^
nAff	0.13^∗∗^	–^a^	–^a^	0.09^∗^	–^a^	–^a^
nPow	–^a^	–^a^	0.07	–^a^	–^a^	-0.10

*N = 539. A single indicator latent variable model is used [χ^*2*^(8) = 5.46, *p* = 0.70], coefficients from standardized solution are shown. nAch, nAff, and nPow refer to the needs of achievement, affiliation, and power, respectively. ^∗^*p* < 0.05; ^∗∗^*p* < 0.01 (one-tailed). ^*a*^Coefficient restricted to zero*.

## Discussion

Complementing research on personality from the trait perspective with a motivational perspective, our study sheds further light on the determinants of networking behavior, in particular the goals that “networkers” seek to attain. We show that the nAch is the most important determinant of networking in general, whereas the nAff and nPow only relate to specific facets of networking.

On a general level, our use of a motivational perspective on personality highlights the instrumental character of networking. While research applying the trait approach highlights that networking is a genuinely social activity, the present study shows that networking is not only determined by social needs (i.e., nAff), but rather serves instrumental goals; it facilitates access to work and career-related resources by means of social contacts. We believe this has two important implications: First, resources obtained by networking are not acquired as an accidental by-product of a genuine social need (i.e., serendipitous networking), but are based on strategic considerations about the “value” of business contacts. Second, our findings that the (nonsocial) nAch is most consistently related to networking shows that networking truly is a career self-management strategy and resembles the relationship between motives and entrepreneurial behavior. The goal of networkers is to excel in their work and career and these people use networking strategically as a means to attain this end.

Also, in line with previous studies (Michael and Yukl, 1993; Forret and Dougherty, 2001; Wolff and Muck, 2009; Wolff and Kim, 2012), this study further corroborates the perspective of differential relationships of personality with specific networking facets. More specifically, we found differential relationships of the social motives nAff and nPow with the functional facet of building, maintaining, and using contacts. Thus, the ability to implement the full range of networking behaviors depends on several distinct determinants and this might not only explain differences in networking behaviors, but also the rarity of highly proficient “networkers.” In addition, support for our hypotheses appears to depend on the structural facet of networking, because we confirmed more of our hypotheses with regard to internal as opposed to external networking, and consistently, relationships are somewhat (i.e., insignificantly) weaker for external networking. This appears to contradict the situational strength hypothesis if we assume that situations within organizations are more strictly governed by norms and role expectations than situations outside organizations. However, note that higher effort that people need to expend for external networking might provide a suitable explanation. If people network to satisfy social needs, this most likely can be accomplished with little effort within organizations. Only if people network to attain excellence in their work or career this might not be fully accomplished within organizations. Career self-management in times of boundaryless careers necessitates an orientation beyond organizational boundaries.

Our findings show that nAch is the most consistent and broad determinant of networking behaviors. Individuals high in nAch seek to attain high performance and occupational success. In this regard, networking is a viable strategy to self-manage individual careers and attain these goals. Our findings also point to how individuals high in nAch succeed. Instead of working harder to obtain higher performance or career success, networking might be a more efficient strategy to reach this goal. The present association of networking with nAch also uncovers a highly important question for future research on networking. Although debated (see e.g., Winter, 2010), McClelland (McClelland and Burnham, 1976; McClelland and Boyatzis, 1982) suggests that nAch determines success only at lower levels of organizational hierarchies, where success is achieved by work on technical tasks. Networking behaviors, which are focused at cooperative, reciprocal relationships, might be an efficient means to obtain resources and success at these lower echelons. Yet, McClelland and coauthors further argue that nPow is the driver to reach top management whenever managers have to work through others and struggles over resources and power are more vigorous. As research on networking has not examined hierarchical level as a moderating variable, this research and the present finding raises the question whether networking yields success only at lower hierarchical levels, but is not a viable strategy to reach top levels of management.

Our study also shows that nAff is positively associated with building contacts, but not maintaining or using contacts. This pattern of relationships between this social need and scales from the functional facet is in line with Wolff and Kim’s (2012) suggestion of decreasing social (and increasing instrumental) concerns from building contacts to maintaining and to using contacts. However, in contrast to our hypothesis, nAff is not related to maintaining contacts, possibly because social concerns are of less importance than we presumed. Individuals high in nAff do not focus their networking behaviors on instrumental goals, that is, they do not focus on maintaining potentially valuable contacts or tapping their contacts’ resources. Rather, nAff drives all kinds of pleasant interactions, but these are not necessarily related to business aspects.

Finally, the nPow is associated with using internal, but not external contacts. We argued that individuals high in nPow overemphasize the instrumental concerns at the cost of social concerns. Individuals high in nPow often break up coalitions and are exploitative in negotiations (McClelland, 1987). These behaviors strongly contrast with maintaining cooperative relations. Our results corroborate Zanzi et al.’s (1991) suggestion that networking is a soft political tactic. In addition, given that networking is a long-term investment into contacts (Wolff and Moser, 2010), it appears questionable whether networking behaviors of individuals high in nPow are effective. Moreover, we found no relationship between nPow and using external contacts. It could be argued that relationships within organizations are characterized by higher longevity and less exchangeability of persons who possess valued resources. Due to these constraints within organizations, people high in nPow might resort to softer political tactics such as networking, whereas concerning external contacts, the consequences to break up coalitions and exploit others might be less serious.

Our study also has practical implications for individuals as well as organizations. Knowledge on individuals’ motives and how they relate to networking might help individuals in developing their networking skills and also trainers in providing customized training. Given that implicit motives operate without awareness, individuals might gain insights as to why they rarely show some behavioral facets of networking. Given a low degree of a particular motive, individuals might have to consciously focus on specific networking facets and actively regulate their networking behaviors. As this conscious focus might deplete resources, individuals should also be aware that they might not be able to permanently perform certain networking behaviors, for example, at a trade fair (Kehr, 2004b). Trainers should bear this knowledge in mind when they focus on improving specific networking skills, as well. Also, organizations that use or develop networking events might benefit from our findings. Our findings show that individuals high in nAch are most likely to network and therefore these organizations might consider using additional incentives to attract individuals with other motivational patterns. In addition, given that not all individuals proactively initiate behaviors aimed at building, maintaining, and using contacts, organizations might consider implementing specific rules or standards to support the entire process of relationship development, for example, by means of goal setting (i.e., make at least two new contacts or one referral).

With regard to limitations, we emphasize that our study is limited to the business domain. As relations between motives and success might be different in other domains, in particular politics (Winter, 2010), we caution scholars to generalize the present findings to other domains. In addition, a possible limitation is our cross-sectional design that yields only weak causal evidence for our hypotheses. Yet, McClelland (1987; McClelland and Franz, 1992) suggests that implicit motives develop early in life, whereas networking behaviors are tied to the entry into the labor force. We therefore argue that it is highly unlikely that reverse causation is responsible for our findings. Finally, and as expected, the effect sizes in our study are small to medium. Yet, they are at about the same size as those reported by Spangler (1992) in his meta-analyses on the relation between implicit motives and standardized questionnaires (i.e., a respondent measure) in a situation where motivational incentives are presumably low (mean *r* = 0.19, *SD* = 0.18, *k* = 108). In addition, as we assessed implicit motives and temporally separated our measures, our effects are probably not inflated by common method bias and likely represent realistic estimates.

Also, as noted by reviewers two additional limitations concern our measure of nPow. First, several networking items do not reflect the nPow, because they mention that a behavior is exhibited to “make new contacts,” “to get confidential advice,” or “out of professional interest” and they rarely appear to address influence or idea selling (see the section “Materials and Methods”). This points to construct coverage of the networking scales and future research might utilize networking items that do not make reference to such proximal goals. Second, Schmalt et al.’s (2000) MMG contains no measure of activity inhibition, a concept that McClelland (1987) used to distinguish personalized power from socialized power. Personalized power (high nPow and low activity inhibition) is often associated with “reckless attempts to show off as being powerful” (McClelland, 1987, p. 302, e.g., speeding, drug abuse), whereas socialized power is associated with more socialized and controlled ways of doing good for others such as holding office or participation in sports and also with reaching top management positions (McClelland, 1987). Note, however, that in the work domain, scholars have utilized and reported evidence for effects of nPow including (McClelland and Boyatzis, 1982; Steinmann et al., 2015) and also excluding (Cornelius and Lane, 1984; Jacobs and McClelland, 1994; Steinmann et al., 2016) activity inhibition from their analyses (see also Spangler and House, 1991). Also note that according to Langens and Schmalt (2008, p. 537), the MMG scales hope of power and fear of power represent the socialized and personalized aspects of power, respectively, to some extent. In this vein, the net motive (i.e., hope of power minus fear of power) might more closely resemble socialized power. Nevertheless, only future research can provide evidence for these assumptions.

To conclude, by adopting theory on implicit motives, we go beyond the lexical trait approach used in prior research and focus on why individuals network. With our focus on implicit goals and desires we find that networking is not simply driven by social goals. Rather, the nAch is the predominant driver of networking behaviors, and only some other facets are driven by other motives.

## Ethics Statement

This study was carried out in accordance to the APA ethical guidelines, that is, all participants were informed about the study aim, duration, and how to obtain information on results. They were informed about whom to contact for any questions about the research or their rights, about their right to leave the survey at any time without any prejudice, and subjects were guaranteed anonymity and confidentiality of their data. As subjects were members of an online panel, they regularly responded to surveys and had experience in study participation. It was therefore deemed that minimal risk was involved and no independent ethical review was conducted.

## Author Contributions

BB contributed to the study design and collected the data. JW contributed to the first draft of the paper. H-GW contributed to the study design, analyzed the data and contributed to the first draft of the paper. All authors contributed to data interpretation and the paper’s revision and refinement.

## Conflict of Interest Statement

The authors declare that the research was conducted in the absence of any commercial or financial relationships that could be construed as a potential conflict of interest.

## Footnotes

1 Classical studies have suggested that a leadership motive pattern of high nPow, low nAff, and high activity inhibition drives these outcomes. However, scholars have questioned these findings for two reasons. First, they are based on different operationalizations of this pattern (McClelland and Boyatzis, 1982; Jacobs and McClelland, 1994). Second, when controlling for main effects of nPow and nAff, their combined interactive effect becomes negligible (Winter, 1987, 1991; Spangler and House, 1991; De Hoogh et al., 2005). We therefore do not elaborate this pattern here (see also discussion on potential limitations of our assessment of nPow in the section “Discussion”).

2 Note that even though composite reliabilities reported at the end of this paragraph are satisfactory, the reliabilities of the six scales are somewhat below conventional standards, albeit comparable to those reported by Sokolowski et al. (2000). In line with the longstanding debate on the reliability of implicit measures (e.g., Atkinson, 1981; Lundy, 1985), Schmalt et al. (2000) attribute this to the heterogeneity of the stimuli used. Further, they report that retest reliabilities of the scales are satisfactory.

## References

[B1] Abele A. E. Andrä M. S. Schute M. (1999). Wer hat nach dem hochschulexamen schnell ein stelle? Erste ergebnisse der erlanger längsschnittstudie (BELA-E) [Who enters a job quickly after graduating? Results of a longitudinal study (BELA-E)]. *Z. Arbeits Organisationspsychol.* 43 95–101. 10.1026//0932-4089.43.2.95

[B2] Allred B. B. Snow C. C. Miles R. E. (1996). Characteristicts of managerial careers in the 21st century. *Acad. Manag. Exec.* 10 17–27. 10.5465/AME.1996.3145316 9465563

[B3] Andrews R. Boyne G. A. Meier K. J. O’Toole L. J. Walker R. M. (2011). Environmental and organizational determinants of external networking. *Am. Rev. Public Adm.* 41 355–374. 10.1177/0275074010382036 28633689

[B4] Arthur M. B. Rousseau D. M. (1996). *The Boundaryless Career.* New York, NY: Oxford University Press.

[B5] Ashton M. C. Lee K. (2001). A theoretical basis for the major dimensions of personality. *Eur. J. Pers.* 15 327–353. 10.1002/per.417 24881884

[B6] Atkinson J. W. (1981). Studying personality in the context of an advanced motivational psychology. *Am. Psychol.* 36 117–128. 10.1037/0003-066X.36.2.117

[B7] Bargh J. A. Chartrand T. L. (1999). The unbearable automaticity of being. *Am. Psychol.* 54 462–479. 10.1037/0003-066X.54.7.462

[B8] Becker T. E. (2005). Potential problems in the statistical control of variables in organizational research: a qualitative analysis with recommendations. *Organ. Res. Methods* 8 274–289. 10.1177/1094428105278021

[B9] Bernerth J. B. Aguinis H. (2016). A critical review and best practice recommendations for control variable usage. *Pers. Psychol.* 69 229–283. 10.1111/peps.12103

[B10] Bilsky W. Schwartz S. H. (2008). Measuring motivations: integrating content and method. *Pers. Indiv. Differ.* 44 1718–1751. 10.1016/j.paid.2008.02.001 19497938

[B11] Blickle G. John J. Ferris G. R. Liu Y. Haag R. Meyer G. (2012). Fit of political skill to the work context: a two-study investigation. *Appl. Psychol.* 61 295–322. 10.1111/j.1464-0597.2011.00469.x

[B12] Blickle G. Witzki A. H. Schneider P. B. (2009). Self-initiated mentoring and career success: a predictive field study. *J. Vocat. Behav.* 74 94–101. 10.1016/j.jvb.2008.10.008

[B13] Bozionelos N. (2003). Intra-organizational network resources: relation to career success and personality. *Int. J. Organ. Anal.* 11 41–56. 10.1108/eb028962

[B14] Brüderl J. Preisendörfer P. (1998). Network support and the success of newly founded businesses. *Small Bus. Econ.* 10 213–225. 10.1023/A:1007997102930

[B15] Callegaro M. Baker R. Bethlehem J. Göritz A. S. Krosnick J. A. Lavrakas P. J. (2014). “Online panel research. History, concepts, applications and a look at the future,” in *Online Panel Research: A Data Quality Perspective*, eds Callegaro M. Baker R. Bethlehem J. Göritz A. S. Krosnick J. A. Lavrakas P. J. (Chichester: Wiley), 1–22. 10.1002/9781118763520

[B16] Casciaro T. Gino F. Kouchaki M. (2014). The contaminating effects of building instrumental ties: how networking can make us feel dirty. *Adm. Sci. Q.* 59 705–735. 10.1177/0001839214554990

[B17] Cohen A. R. Bradford D. L. (1989). Influence without authority: on the social networks of managers. *Organ. Dyn.* 17 5–17. 10.1016/0090-2616(89)90033-8

[B18] Collins C. J. Hanges P. J. Locke E. A. (2004). The relationship of achievement motivation to entrepreneurial behavior: a meta-analysis. *Hum. Perform.* 17 95–117. 10.1207/S15327043HUP1701_5

[B19] Collins C. J. Clark K. D. (2003). Strategic human resource practices, top management team social networks, and firm performance: the role of human resource practices in creating organizational competitive advantage. *Acad. Manage. J.* 46 740–751. 10.2307/30040665

[B20] Cornelius E. T. I. Lane F. B. (1984). The power motive and managerial success in a professionally oriented service industry organization. *J. Appl. Psychol.* 69 32–39. 10.1037/0021-9010.69.1.32

[B21] Chuzmir L. H. Azevedo A. (1992). Motivation needs of sampled fortune-500 CEOs: relations to organization outcomes. *Percept. Motor Skill.* 7 595–612. 10.2466/pms.1992.75.2.595 1408626

[B22] De Hoogh A. H. B. Den Hartog D. N. Koopman P. L. Thierry H. van den Berg P. T. van der Weide J. G. (2005). Leader motives, charismatic leadership, and subordinates work attitude in the profit and voluntary sector. *Leadersh. Q.* 16 17–38. 10.1016/j.leaqua.2004.10.001

[B23] de Janasz S. C. Forret M. L. (2008). Learning the art of networking: a critical skill for enhancing social capital and career success. *J. Manage. Educ.* 32 629–650. 10.1177/1052562907307637

[B24] Ferris G. R. Blickle G. Schneider P. B. Kramer J. Zettler J. Solga J. (2008). Political skill construct and criterion-related validation: a two-study investigation. *J. Manag. Psychol.* 23 744–771. 10.1108/02683940810896321

[B25] Ferris G. R. Treadway D. C. Kolodinsky R. W. Hochwarter W. A. Kacmar C. J. Douglas C. (2005). Development and validation of the political skill inventory. *J. Manag.* 31 126–152. 10.1177/0149206304271386

[B26] Ferris G. R. Treadway D. C. Perrewe P. L. Brouer R. L. Douglas C. Lux S. (2007). Political skill in organizations. *J. Manag.* 33 290–320. 10.1177/0149206307300813

[B27] Forret M. L. Dougherty T. W. (2001). Correlates of networking behavior for managerial and professional employees. *Group Organ. Manag.* 26 283–311. 10.1177/1059601101263004

[B28] Forret M. L. Dougherty T. W. (2004). Networking behaviors and career outcomes: differences for men and women? *J. Organ. Behav.* 25 419–437. 10.1002/job.253

[B29] French E. G. Lesser G. S. (1964). Some characteristics of the achievement motive in women. *J. Abnorm. Soc. Psychol.* 68 119–128. 10.1037/h0041417 14117962

[B30] Gibson C. Hardy J. H. I. Buckley R. M. (2014). Understanding the role of networking in organizations. *Career Dev. Int.* 19 146–161. 10.1108/CDI-09-2013-0111

[B31] Goldberg L. R. (1990). An alternative “description of personality”: the big-five factor structure. *J. Pers. Soc. Psychol.* 59 1216–1229. 10.1037/0022-3514.59.6.1216 2283588

[B32] Hall D. T. (1996). Protean careers of the 21st century. *Acad. Manag. Exec.* 10 8–16. 10.5465/AME.1996.3145315

[B33] Heckhausen H. (1968). *The Anatomy of Achievement Motivation.* New York, NY: Academic Press.

[B34] House R. J. Spangler W. D. Woycke J. (1991). Personality and charisma in the U.S. presidency: a psychological theory of leader effectiveness. *Adm. Sci. Q.* 36 364–396. 10.5465/AMBPP.1990.4978722

[B35] Huang J. L. Curran P. G. Keeney J. Poposki E. M. DeShon R. P. (2012). Detecting and deterring insufficient effort responding in surveys. *J. Bus. Psychol.* 27 99–114. 10.1007/s10869-011-9231-8

[B36] Jacobs R. L. McClelland D. C. (1994). Moving up the corporate ladder: a longitudinal study of the leadership motive pattern and managerial success in women and men. *Consult. Psychol. J.* 46 1031–1067. 10.1037/1061-4087.46.1.32

[B37] Jöreskog K. Sörbom D. (2005). *LISREL 8.72.* Lincolnwood, IL: Scientific Software International.

[B38] Kehr H. M. (2004a). Implicit/explicit motive discrepancies and volitional depletion among managers. *Pers. Soc. Psychol. Bull.* 30 315–327. 10.1177/0146167203256967 15030623

[B39] Kehr H. M. (2004b). Integrating implicit motives, explicit motives, and perceived abilities. the compensatory model of work motivation and volition. *Acad. Manag. Rev.* 29 479–499. 10.5465/AMR.2004.13670963

[B40] King Z. (2004). Career self-management: its nature, causes and consequences. *J. Vocat. Behav.* 65 112–133. 10.1016/S0001-8791(03)00052-6 23796465

[B41] Kotter J. P. (1982). What effective general managers really do. *Harv. Bus. Rev.* 60 156–167. 10298805

[B42] Kuipers M. A. C. T. Scheerens J. (2006). Career competencies for the modern career. *J. Career Dev.* 32 303–319. 10.1177/0894845305283006

[B43] Lambert T. A. Eby L. T. Reeves M. P. (2006). Predictors of networking intensity and network quality among white collar job seekers. *J. Career Dev.* 32 351–365. 10.1177/0894845305282767

[B44] Lang J. W. B. Zettler I. Ewen C. Hülsheger U. R. (2012). Implicit motives, explicit traits, and task and contextual performance at work. *J. Appl. Psychol.* 97 1201–1217. 10.1037/a0029556 22867444

[B45] Langens T. A. Schmalt H.-D. (2008). “Motivational traits: new directions and measuring motives with the multi-motive grid (MMG),” in *The Sage Handbook of Personality Theory and Assessment*, eds Boyle G. J. Saklofske G. Matthew D. H. (London: Sage), 523–544.

[B46] Langner C. A. Winter D. G. (2001). The motivational basis of concessions and compromise: archival and laboratory studies. *J. Pers. Soc. Psychol.* 81 711–727. 10.1037/0022-3514.81.4.711 11642356

[B47] Lawrence S. Jordan P. (2009). Testing an explicit and implicit measure of motivation. *Int. J. Organ. Anal.* 17 103–120. 10.1108/19348830910948959

[B48] Lundy A. (1985). The reliability of the thematic apperception test. *J. Pers. Assess.* 49 141–145. 10.1207/s15327752jpa4902_6 16367479

[B49] Luthans F. Rosenkrantz S. A. Hennessey H. W. (1985). What do successful managers really do? An observation study of managerial activities. *J. Appl. Behav. Sci.* 21 255–270. 10.1177/002188638502100303

[B50] MacCallum S. Y. Forret M. L. Wolff H.-G. (2014). Internal and external networking behavior: an investigation of relationships with affective, continuance, and normative commitment. *Career Dev. Int.* 19 595–614. 10.1108/CDI-08-2013-0101

[B51] Malhotra N. (2008). Completion time and response order effects in web surveys. *Public Opin. Q.* 72 914–934. 10.1093/poq/nfn050

[B52] McAdams D. P. Olson B. D. (2010). Personality development: continuity and change over the life course. *Annu. Rev. Psychol.* 61 517–542. 10.1146/annurev.psych.093008.100507 19534589

[B53] McClelland D. C. (1987). *Human Motivation.* Cambridge: Cambridge University Press.

[B54] McClelland D. C. Boyatzis R. E. (1982). Leadership motive pattern and long-term success in management. *J. Appl. Psychol.* 67 737–743. 10.1037/0021-9010.67.6.737

[B55] McClelland D. C. Burnham D. H. (1976). Power is the great motivator. *Harv. Bus. Rev.* 54 100–110. 12545928

[B56] McClelland D. C. Franz C. E. (1992). Motivational and other sources of work accomplishments in mid-life: a longitudinal study. *J. Pers.* 60 679–707. 10.1111/j.1467-6494.1992.tb00270.x

[B57] McClelland D. C. Koestner R. Weinberger J. (1989). How do self-attributed and implicit motives differ? *Psychol. Rev.* 96 690–702. 10.1037/0033-295X.96.4.690 24367351

[B58] McCrae R. R. Costa P. T. J. (1997). Personality trait structure as a human universal. *Am. Psychol.* 52 509–516. 10.1037/0003-066X.52.5.509 9145021

[B59] Michael J. Yukl G. (1993). Managerial level and subunit function as determinants of networking behavior in organizations. *Group Organ. Manag.* 18 328–351. 10.1177/1059601193183005

[B60] Ng T. W. H. Feldman D. C. (2010). The effects of organizational embeddedness on development of social capital and human capital. *J. Appl. Psychol.* 95 696–712. 10.1037/a0019150 20604589

[B61] Ng T. W. H. Feldman D. C. (2011). Locus of control and organizational embeddedness. *J. Occup. Organ. Psychol.* 84 173–190. 10.1348/096317910X494197

[B62] Nunnally J. C. (1978). *Psychometric Theory.* New York, NY: McGraw-Hill.

[B63] Pentland A. (2012). The new science of building great teams. *Harv. Bus. Rev.* 90 60–69.23074865

[B64] Pfeffer J. (1981). *Power in Organizations.* Cambridge, MA: Ballinger.

[B65] Podsakoff P. M. MacKenzie S. B. Podsakoff N. P. (2012). Sources of method bias in social science research and recommendations on how to control it. *Annu. Rev. Psychol.* 63 539–569. 10.1146/annurev-psych-120710-100452 21838546

[B66] Porter C. M. Woo S. E. (2015). Untangling the networking phenomenon: a dynamic psychological perspective on how and why people network. *J. Manag.* 41 1477–1500. 10.1177/0149206315582247

[B67] Porter C. M. Woo S. E. Campion M. A. (2016). Internal and external networking differentially predict turnover through job embeddedness and job offers. *Pers. Psychol.* 69 635–672. 10.1111/peps.12121

[B68] Rauch A. Frese M. (2007). Let’s put the person back into entrepreneurship research: a meta-analysis on the relationship between business owners’ personality traits, business creation, and success. *Eur. J. Work Organ. Psychol.* 16 353–385. 10.1080/13594320701595438

[B69] Schmalt H.-D. Sokolowski K. Langens T. A. (2000). *Multi-Motiv-Gitter (MMG).* Frankfurt: Harcourt Test Services.

[B70] Schultheiss O. C. Brunstein J. C. (2001). Assessment of implicit motives with a research version of the TAT: picture profiles, gender differences, and relations to other personality measures. *J. Pers. Assess.* 77 71–86. 10.1207/S15327752JPA7701_05 11562105

[B71] Schultheiss O. C. Brunstein J. C. (2002). Inhibited power motivation and persuasive communication: a lens model analysis. *J. Pers.* 70 553–582. 10.1111/1467-6494.05014 12095191

[B72] Schultheiss O. C. Strasser A. Rösch A. G. Kordik A. Graham S. C. C. (2012). “Motivation,” in *Encyclopedia of Human Behavior*, 2nd Edn, ed. Ramachandran V. S. (Oxford: Elsevier), 650–656. 10.1016/B978-0-12-375000-6.00238-X

[B73] Semrau T. Sigmund S. (2012). Networking ability and the financial performance of new ventures: a mediation analysis among younger and more mature firms. *Strateg. Entrep. J.* 6 335–354. 10.1002/sej.1146

[B74] Sokolowski K. Schmalt H.-D. Langens T. A. Puca R. M. (2000). Assessing achievement, affiliation, and power motives all at once: the multi-motive grid (MMG). *J. Pers. Assess.* 74 126–145. 10.1207/S15327752JPA740109 10779937

[B75] Spangler W. D. (1992). Validity of questionnaire and TAT measures of need for achievement: two meta-analyses. *Psychol. Bull.* 112 140–154. 10.1037/0033-2909.112.1.140

[B76] Spangler W. D. House R. J. (1991). Presidential effectiveness and the leadership motive profile. *J. Appl. Psychol.* 60 439–455. 10.1037/0022-3514.60.3.439

[B97] Spector P. E. Zapf D. Chen P. Y. Frese M. (2000). Why negative affectivity should not be controlled in job stress research: don’t throw out the baby with the bath water. *J. Organ. Behav.* 21 79–95. 10.1002/(SICI)1099-1379(200002)21:1<79::AID-JOB964>3.0.CO;2-G

[B77] Steinmann B. Dörr S. L. Schultheiss O. C. Maier G. W. (2015). Implicit motives and leadership performance revisited: what constitutes the leadership motive pattern? *Motiv. Emot.* 39 167–174. 10.1007/s11031-014-9458-6

[B78] Steinmann B. Ötting S. K. Maier G. W. (2016). Need for affiliation as a motivational add-on for leadership behaviors and managerial success. *Front. Psychol.* 7:1972. 10.3389/fpsyg.2016.01972 28066295 PMC5177659

[B79] Sturges J. J. L. (2008). All in a day’s work? Career self-management and the management of the boundary between work and non-work. *Hum. Resour. Manag. J.* 18 118–134. 10.1111/j.1748-8583.2007.00054.x

[B80] Thompson J. A. (2005). Proactive personality and job performance: a social capital perspective. *J. Appl. Psychol.* 90 1011–1017. 10.1037/0021-9010.90.5.1011 16162073

[B81] UNESCO Institute for Statistics (2012). *International Standard Classification of Education.* Montreal, QC: UNESCO Institute for Statistics.

[B82] Van Hoye G. Van Hoft E. A. J. Lievens F. (2009). Networking as a job search behavior: a social network perspective. *J. Occup. Organ. Psychol.* 82 661–682. 10.1348/096317908X360675

[B83] Wanberg C. R. Kanfer R. Banas G. T. (2000). Predictors and outcomes of networking intensity among unemployed job seekers. *J. Appl. Psychol.* 85 491–503. 10.1037//0021-9010.85.4.401 10948794

[B84] Winter D. G. (1987). Leader appeal, leader performance, and the motive profiles of leaders and followers: a study of American presidents and elections. *J. Pers. Soc. Psychol.* 52 196–202. 10.1037/0022-3514.52.1.196

[B85] Winter D. G. (2007). The role of motivation, responsibility, and integrative complexity in crisis escalation: comparative studies of war and peace crises. *J. Pers. Soc. Psychol.* 92 920–937. 10.1037/0022-3514.92.5.920 17484613

[B86] Winter D. G. (2010). Why achievement motivation predicts success in business but failure in politics: the importance of personal control. *J. Pers.* 78 1637–1668. 10.1111/j.1467-6494.2010.00665.x 21039527

[B87] Winter D. G. (1991). A motivational model of leadership: predicting long-term management success from TAT measures of power motivation and responsibility. *Leadership Quart.* 2 67–80. 10.1016/1048-9843(91)90023-U

[B88] Winter D. G. John O. P. Stewart A. J. Klohnen E. C. Duncan L. E. (1998). Traits and motives: toward an integration of two traditions in personality research. *J. Pers. Soc. Psychol.* 105 230–250. 10.1037/0033-295X.105.2.230 9577238

[B89] Witt P. (2004). Entrepreneurs’ networks and the success of start-ups. *Entrep. Reg. Dev.* 16 391–412. 10.1080/0898562042000188423

[B90] Wolff H.-G. Kim S. (2012). The relationship between networking behaviors and the big five personality factors. *Career Dev. Int.* 17 43–66. 10.1108/13620431211201328

[B91] Wolff H.-G. Moser K. (2006). Entwicklung und validierung einer networkingskala [Development and validation of networking scale]. *Diagnostica* 52 161–180. 10.1026/0012-1924.52.4.161

[B92] Wolff H.-G. Moser K. (2009). Effects of networking on career success: a longitudinal study. *J. Appl. Psychol.* 94 196–206. 10.1037/a0013350 19186904

[B93] Wolff H.-G. Moser K. (2010). Do specific types of networking predict specific mobility outcomes? A two-year prospective study. *J. Vocat. Behav.* 77 238–245. 10.1016/j.jvb.2010.03.001

[B94] Wolff H.-G. Muck P. M. (2009). Persönlichkeit und networking: eine analyse mittels interpersonalem circumplex [Personality and networking: an analysis using the interpersonal circumplex]. *Z. Personalpsychol.* 8 106–116. 10.1026/1617-6391.8.3.106

[B95] Wolff H.-G. Rahm C. Forret M. L. (2011). Adaptation of a German multidimensional networking scale into English. *Eur. J. Psychol. Assess.* 27 244–250. 10.1027/1015-5759/a000070

[B96] Zanzi A. Arthur M. B. Shamir B. (1991). The relationship between career concerns and political tactics in organizations. *J. Organ. Behav.* 12 219–233. 10.1002/job.4030120305

